# ROS, Cell Senescence, and Novel Molecular Mechanisms in Aging and Age-Related Diseases

**DOI:** 10.1155/2016/3565127

**Published:** 2016-05-10

**Authors:** Pierpaola Davalli, Tijana Mitic, Andrea Caporali, Angela Lauriola, Domenico D'Arca

**Affiliations:** ^1^Department of Biomedical, Metabolic and Neural Sciences, University of Modena & Reggio Emilia, 41125 Modena, Italy; ^2^Bristol Heart Institute, University of Bristol, Bristol BS2 8HW, UK; ^3^University/BHF Centre for Cardiovascular Science, The Queen's Medical Research Institute, 47 Little France Crescent, Edinburgh EH16-4TJ, UK; ^4^Istituto Nazionale di Biostrutture e Biosistemi, 00136 Roma, Italy

## Abstract

The aging process worsens the human body functions at multiple levels, thus causing its gradual decrease to resist stress, damage, and disease. Besides changes in gene expression and metabolic control, the aging rate has been associated with the production of high levels of Reactive Oxygen Species (ROS) and/or Reactive Nitrosative Species (RNS). Specific increases of ROS level have been demonstrated as potentially critical for induction and maintenance of cell senescence process. Causal connection between ROS, aging, age-related pathologies, and cell senescence is studied intensely. Senescent cells have been proposed as a target for interventions to delay the aging and its related diseases or to improve the diseases treatment. Therapeutic interventions towards senescent cells might allow restoring the health and curing the diseases that share basal processes, rather than curing each disease in separate and symptomatic way. Here, we review observations on ROS ability of inducing cell senescence through novel mechanisms that underpin aging processes. Particular emphasis is addressed to the novel mechanisms of ROS involvement in epigenetic regulation of cell senescence and aging, with the aim to individuate specific pathways, which might promote healthy lifespan and improve aging.

## 1. Introduction

The reduced rate of birth and mortality is the motive of the older population growth in western industrialized countries, where advanced age remains the fundamental risk factor for most chronic diseases and functional deficits. As an example, it is estimated that the individuals of age 65 and above in the USA will reach 20% by 2030, while they constituted 12.4% in 2004 [1]. Human aging is developed from such an accumulation of physical, environmental, and social factors that the definition of the molecular mechanisms that trigger the aging means a difficult task. Some theories associate various factors with aging rate, as changes of metabolic control [2] and gene expression patterns [3] and production of high levels of Reactive Oxygen Species (ROS) [4]. Low ROS level has been, instead, associated with lengthening of organismal lifespan [5]. Current studies aim at deepening how cell senescence process, so far experimented* in vitro*, may be extended to* in vivo* studies. Increasing evidence for causal role of cell senescence has been demonstrated in age-related dysfunctions and pathologies [6]. Senescent cells proliferate in aging, as a stress response primed by a number of “counting mechanisms,” like telomeres shortening, DNA damage accumulation, abnormal oncogenes activities, metabolic alterations, and excessive ROS generation [7]. These mechanisms cause cell proliferating arrest and generate features, as constitutive production of high ROS levels, critical for the senescent phenotype maintenance. Despite increasing modestly, as a number, the senescent cells are implicated in age-related diseases promotion, through the restriction of the regenerative pool of the tissue stem cells [8]. Some observations indicate that senescent cells do not necessarily induce mechanisms that promote aging and can be efficiently removed from the human body [9]. The general consensus on cellular damage accumulation, as aging initial event, suggests that cell senescence process is a major question regarding biological and clinical aging aspects [10].

Here, we review evidences on novel molecular mechanisms of the “ROS signaling” during aging and related pathologies, because they suggest a way of promoting healthy lifespan and improve human aging.

## 2. ROS Physioma Homeostasis

The ROS physioma is a family of highly reactive molecules which includes free oxygen radicals, like superoxide anion (O_2_^•−^), hydroxyl radical (OH^•^), and nonradical oxygen derivatives, like the stable hydrogen peroxide (H_2_O_2_). The superoxide radicals react to form other ROS, namely, hydrogen peroxides and hydroxyl radicals, and interconvert with reactive nitrogen species (RNS), which generate effects similar to ROS [11]. The inefficient electron transfer in mitochondrial respiratory chain is believed to be a main ROS source, among diverse possible enzymatic and nonenzymatic sources [12]. Increased expression of catalase and peroxiredoxin-1 molecules are considered as OS markers. The family comprises seven transmembrane members, namely, Nox1–5 [13–15] and Duox1-2 [16]. ROS are generated by oxygen metabolism (i.e., cellular respiration) in all the cells that utilize oxygen, as inevitable consequence of aerobic life, and may derive from exogenous metals, recycling of redox compounds, radiation, chemotherapeutic agents, carcinogens (estrogenic molecules), and other dietary and environmental means. Generally, the ROS increasing levels cause nonlinear cellular responses [17]. A fine balance between oxidant-antioxidant mechanisms leads to continuous modulation of ROS production, location, and inactivation, in both physiological and pathological conditions. Endogenous antioxidants, like the enzymes of catalase family, glutathione group, thioredoxin-related group, and superoxide dismutase [18], together with exogenous antioxidant as reduced glutathione [19], carotenoids, and vitamins C and E, constitute the indispensable ROS detoxifying system. Nevertheless, imbalance of redox homeostasis may occur, usually in favor of oxidants, so that ROS shift from physiological to potentially harmful levels, named oxidative and nitrosative stress (OS/NS). Increased expression of catalase and peroxiredoxin 1 molecules are considered as OS markers [20–22].

### 2.1. ROS Measurement Techniques

ROS are so highly variable and freely diffusible molecules that the detection of ROS and antioxidants, to obtain a picture of the cellular redox status, still represents a challenge. We stress some specific points and sensitive methods that are subjected to continuous improvement. Probes and antibodies have been developed to recognize oxidative damage by ROS/RNS [23–25]. The tools allow revealing antioxidant enzymes [26] and a variety of oxidative products, as lipid peroxidation products, protein carbonyls [27], oxidized DNA products [28], and nitrotyrosine [29]. Combinations of diverse approaches will prove essential for understanding ROS involvement in aging and age-related diseases [30]. An innovative method simultaneously assesses glutathione, hydrogen peroxide, and superoxide levels in a single cell, together with cell viability alterations, thus allowing for defining both oxidant-antioxidant balance and cell death, after the administration of a specific stimulus [31]. A wide range of pathways and molecular mechanisms that involve ROS suggests determining the redox state of thiols in ROS targets, which compose the “cellular oxidative interface” [32, 33]. ROS oxidize specific protein residues of cysteine into sulfenic acid, reversibly. This molecule functions as OS/NS sensor within enzymes and transcriptional regulatory factors and may allow priming the routes of the versatile ROS action [34–36].

### 2.2. ROS Functions

The increasing comprehension of mechanisms, underlying the oxidant milieu of the cell, shows ROS as signaling molecules, besides metabolic byproducts. They act in a myriad of pathways and networks, mediated by hormones, which ranges from protein phosphorylation to transport systems, for example. ROS do not influence single steps of multistep processes; rather, they influence all the steps at the same time, by reacting with several compounds and taking part in several redox reactions. Depending on ROS concentration, molecular species, and subcellular localization, cell components and signaling pathways are affected positively or negatively. ROS levels are believed to be a “redox biology” that regulates physiological functions, including signal transduction, gene expression, and proliferation. “Redox biology,” rather than OS, has been proposed to underlie both physiological and pathological events [37]. Data in the literature on slow and constant ROS increases have to be integrated with data on fast and stepwise ROS increases, typical of signaling events, which deliver messages among cellular compartments. Questions related to ROS dynamics and specificity, as the effects of their waves of concentration on networks with other signaling pathways, are investigated in single cells and across different cells. Proteins are the major target of ROS/RNS signaling and undergo reversible or irreversible modifications of their functions, which result in cell death, growth arrest, and transformation. The modulation of the reversible oxidation of redox-sensitive proteins plays basic roles in sensing and transducing the oxygen signal. Receptor-dependent or nondependent tyrosine kinases, AMP-activated protein kinases, adaptor protein p66SHC, and transcription factors as FOXO (forkhead homeobox type O), Nrf2 (nuclear factor E2-related factor 2), p53 (tumor suppressor 53), NF-*κ*B (nuclear factor kappa B), AP-1 (activator protein-1), HIF-1a (hypoxia inducible factor-1a), PPAR*γ* (peroxisome proliferator-activated receptor gamma), and *β*-catenin/Wnt signaling are listed in Table 1 [38–81]. ROS mediate* in vitro* response towards intra- and extracellular conditions, such as growth factors, cytokines, nutrients deprivation, and hypoxia, which regulate cell proliferation, differentiation, and apoptosis, besides being important cancer hallmarks [82]. Intrinsic and extrinsic factors control ROS regulation on cellular self-renewal, quiescence, senescence, and apoptosis, during the* in vivo* tissues homeostasis and repair [83] and in ROS induction of stem cells proliferation and differentiation. ROS act as a rheostat, which senses and translates environmental cues in stem cells response, thus balancing cellular output (function) with cellular input (nutrients, cytokines). The stem cells may undergo exhaustion depending on ROS levels [84]. Mitochondrial ROS may activate an adaptive response (mitohormesis), which, as defensive mechanism, promotes health to extend the lifespan through diseases prevention and delay [5, 85]. ROS is integral in the development of physiopathologic events like mitochondrial death signaling [86] and autophagy [87], besides inflammation and infection [55, 88], in which they impart immunological changes. High ROS levels are generated by professional cells (lymphocytes, granulocytes, and phagocytes) in defense against microbes [89, 90]. Differently, any event which contributes to chronic OS or NS, through its increased generation or defective detoxification, dysregulates signaling networks, alters lipids and protein and nucleic acids, and activates mechanisms to face the changes. ROS overproduction hampers damaged nuclear and mitochondrial DNA repair, at multiple steps, contributing to cell genomic instability [91]. ROS are recognized as key modulators in processes that accumulate oxidized molecules chronically, as diabetes, cardiovascular diseases, atherosclerosis, hypertension, ischemia, reperfusion injury, neurodegeneration, and rheumatoid arthritis [17]. Also, ROS participate in cancer development through their effects on cellular proliferation, mutagenesis, and apoptosis inhibition [56]. The cross talk between ROS, p53, and NF-*κ*B plays crucial roles in tumorigenesis. OS is allied with energy metabolism to stimulate the growth of cells transformed by oncogenes or tumor suppressors [92–94]. The deregulated ROS productions in cancer cells and the consequent constitutive OS may cause the cellular invasive phenotype [57].

Although ROS functions remain difficult to investigate, multiple pharmacological investigations are in progress to maintain ROS homeostasis through both OS decrease and antioxidant defense increase [95, 96].

## 3. ROS in Aging and Age-Related Diseases

Poor knowledge of basic processes in aging interferes with interventions to prevent or delay age-related pathologies, like diabetes, cardiovascular disorders, neurodegenerative disorders, and cancer, which, consequently, impact human independence, general wellbeing, and morbidity [97–99]. Recently, interest has been focused on stem cells, because their decline impairs tissues homeostasis maintenance, leading to the organism weakening and the age-related diseases [84]. Aging mechanisms have been collected into two classes. The first class presents aging as genetically programmed by developmental processes, like the cell senescence, the neuroendocrine alterations, and the immunological alterations. The second class presents aging caused by random damage, that is, accumulation of somatic mutations and OS. The separation between the classes is no longer considered clear, because pathways involved in aging often share features with specific diseases [100]. The genetic heredity contributes no more than 3% to aging, while epigenetic processes and posttranslational processes imprint a significantly different aging rate among diverse populations, as well as among diverse anatomical sites of a single organism. In the onset of aging, telomere erosion, OS, and cell senescence are crucial events that originate from the disorganized homeostasis of cell metabolism. For example, mitochondria-nucleus interplay [101] and alterations of mitochondrial homeostasis drive age-dependent modifications [102, 103]. Ineffective ROS control on mitochondrial supercomplexes causes ROS signaling alteration, thus mediating cell stress responses towards age-dependent damage [104]. A progressive ROS scavengers decrease shifts aged cells towards a prooxidant status [105, 106]. In parallel, all the suggested methods to prolong lifespan, as caloric restriction and increased activity of SIRT1, share the OS reduction effect [107]. It is known that chronic muscular exercise protects older persons from damage caused by OS and reinforces their defenses against it. On the other hand, acute exercise increases ROS production and damage from ROS [108]. High levels of mitochondrial ROS contribute to aging of genetically modified animals, in a mechanistic way. Superoxide dismutase-deficient animals, SOD1- [109] and SOD3-deficient animals [110], and p66SHC-deficient animals show mitochondrial dysfunctions that generate oxidative damage and related phenotypes, resembling premature aging features. Similarly, mice that overexpress mitochondrial catalase counteract oxidative damage and live longer. The incidence of age-related diseases and pathologies in animal models, after they have been submitted to disparate patterns, suggests that OS influences old age aspects significantly [111]. The observations have been extended to humans, even if rate and distribution of mitochondrial mutations may deviate from animals. The conclusions regarding OS effect on aging in animals from mitochondrial genetic manipulations are still conflicting. SOD+/− mice have reduced ROS detoxifying ability and high ROS level, while they exhibit a quite normal lifespan. OS effect on worms' lifespan depends on where ROS are produced: high mitochondrial or cytoplasmic levels are associated with increased and decreased lifespan, respectively [109, 112]. It remains to define whether models' longevity is entirely associated with response to OS, because their lifespan is not affected by modulation of the antioxidant defense. The complex genetic manipulation of the models might weaken their support to the “OS theory of aging.” Interventions to ROS lowering, by both scavenging free radicals and enhancing antioxidant defenses, are widely proposed as an antiaging strategy. However, positive association between supplementation with pharmacological or natural compounds and health beneficial effects has not been evidenced. Some antioxidants may be eventually useless or even harmful [113, 114]. Moreover, a number of ROS-independent mitochondrial dysfunctions appear so involved in aging that doubts arise that OS is the most concrete contributor to fuel aging [115]. Based on the consideration that mitochondrial DNA (mtDNA) is a precise marker to detect total mitochondrial OS, methods have been developed to measure mtDNA replication defects and the oxidative damage level, simultaneously. The errors in mtDNA replication and repair, which accumulate through clonal expansion in advanced age, result in a major source of mtDNA mutations, rather than the errors acquired through ROS-dependent vicious cycles [116]. Summarizing, ROS are involved in elderly lesions that concern (i) DNA insufficiency, which is partly responsible for premature aging and apoptosis [117]; (ii) RNA involvement in the onset of chronic-degenerative diseases [118]; (iii) nuclear lamins that participate in cell proliferation and longevity [119]. The variations of speed and quality in the aging of each organism may reflect the peculiar alterations that have been accumulated in DNA, proteins, and lipids [120], following the organism exposition to chronic stressors. Low ROS levels improve the defense mechanisms by inducing adaptive responses, which contributes to stress resistance and longevity, while high ROS levels induce insufficient adaptive responses, which may contribute to aging onset and progression [121].

In conclusion, accumulated mutations, decreased mitochondrial energy metabolism, and increased OS may significantly contribute to the human aging and the related diseases.

## 4. ROS-Dependent Epigenetic Modifications

Intra- and extracellular environments change hereditary characters at the epigenetic level, without altering genes sequence [122]. The interplay between modified histones, DNA methylation, regulator noncoding RNAs, and other reversible processes constitutes the epigenetic machinery that regulates genes transcription and expression [123]. The epigenetic modulation provides the essential and flexible interface between organism and environment, which is essential for all the cell functions. The extent to which epigenome has shaped, and might shape, human populations over generations is investigated by an International Human Epigenome Consortium (http://www.ihec-epigenomes.org/). Both long- and short-acting stimuli lead to epigenetic effects that result in 13 being long-term (heritable) or short-term (nonheritable), respectively. These features suggest epigenetic modifications as more attractive target for therapeutic interventions in humans than genetic modification, throughout the entire life [124]. ROS operate modifications on histone and DNA, by acting in interconnected epigenetic phases, during mitochondrial and nuclear DNA regulation [125, 126]. A clinical example of ROS-dependent epigenetic modifications is demonstrated in “nonalcoholic fatty liver” disease. The pathology represents the most common cause of chronic liver disease in western countries and affects one-third of the population. Altered redox mechanisms mediate the link between increased accumulation of triglycerides in hepatocytes and epigenetic modifications that are recognized as crucial factors in the pathophysiology of this disease [127]. About the basic mechanisms of ROS action, Afanas'ev proposes that ROS might cause epigenetic activation and repression, by acting like nucleophilic compounds, which accelerate and decelerate hydrolysis and esterification reactions. The hypothesis suggests a ROS role different from free radicals, because the last molecules cause an irreversible damage of the compounds with which they react [128].

### 4.1. ROS-Induced DNA Methylation

Usually, condensed chromatin structure (heterochromatin) is associated with genes repression by hypomethylation processes, while open chromatin (eu-chromatin) is associated with genes activation by acetylation processes [129]. The epigenetic marking modulates the genes expression by altering the electrostatic nature and the protein binding affinity of chromatin. DNA methylation causes gene silencing through inhibiting the transcriptional activators access to the target binding sites, or through activating the methyl-binding protein domains. The last function interacts with histone deacetylases and promotes chromatin condensation into transcriptionally repressive conformations. Hypo- and hypermethylation stages occur consecutively, indicating how DNA methylation and the correlate mechanisms of DNA binding are complex. ROS-dependent modifications are related to DNA methylation and demethylation, directly or indirectly. The NF-*κ*B binding to DNA, which is methylation dependent, results in being altered in SOD (Cu/Zn)-deficient mice. The observation associates ROS-dependent modifications with altered methylation processes, although indirectly, and suggests that modifications linked to altered redox mechanisms may fit into cell signaling pathways [130]. Also, the oxidation of deoxy-guanine of CpG nucleotides to 8-hydroxy-2′-deoxyguanosine (8-OHdG) is believed to be a surrogate marker of oxidative damage, in various human diseases [131]. The 8-OHdG adducts interfere with DNA restriction nucleases and DNA methyl transferases (DNMT), thus altering transcription factors binding to DNA and causing general DNA hypomethylation.* In vitro* [132] and* in vivo* [133] studies demonstrate that ROS induce general genome hypomethylation and specific DNA promoters hypomethylation, via the DNMT upregulation and the DNMT complexes generation. Moreover, recent studies show that a ROS-mediated pathway causes repression of the protein kinase C epsilon gene, through its promotor methylation. The events are important in heart hypoxia,* in utero*, which leads to heightened heart vulnerability to ischemic injury, later in people's life [134].

### 4.2. ROS and DNA Methylation in Aging and Age-Related Diseases

**Figure 1:**
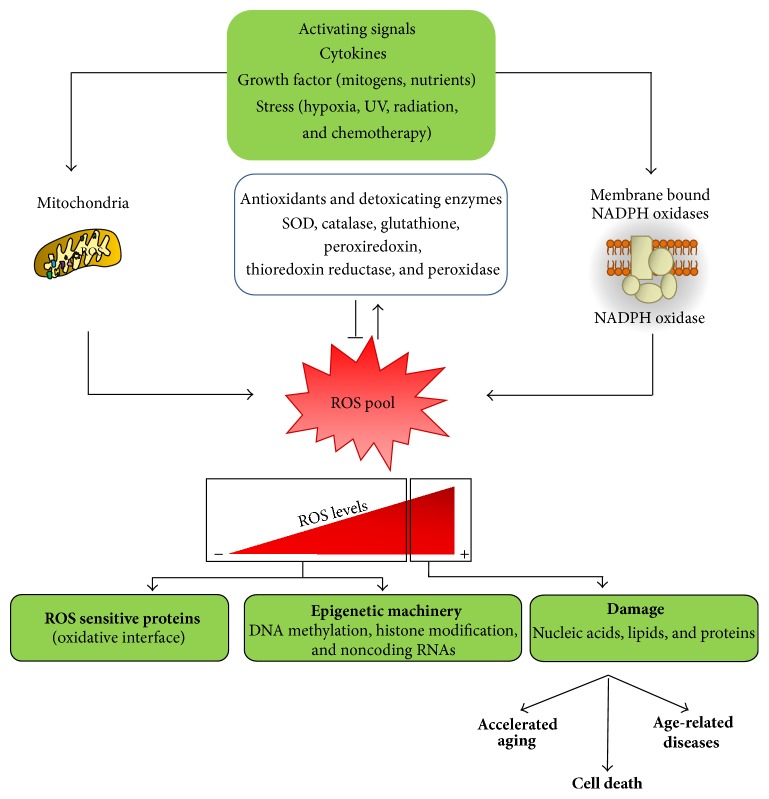
Schematic representation of ROS signaling in physiological and pathological conditions. Low and medium ROS levels produced by mitochondria and NADPH oxidase activate cell ROS sensitive proteins and epigenetic machinery. High ROS level causes nucleic acids, lipid, and proteins damage possibly involved in accelerated aging, cell death, and age-related diseases.

Starting from the observation that both defective genome and DNA repair processes promote phenotypes of premature aging, the “aging epigenetics” has been developed as emerging discipline, which concerns genes and processes impacting aging (Figure 1) [135]. ROS effects on epigenetic mechanisms have been discussed as cause and consequence of aging and age-related DNA modifications [128]. Recent studies demonstrate that global DNA hypomethylation is deeply included in aging gene expression [136], and, at the same time, cancer is the age-related disease that shows the most significant effects of ROS-dependent DNA methylation [137]. Tumor progression is induced by general hypomethylation of the DNA and hypermethylation of tumor suppressor genes that lead to aberrant genes expression [138–140]. Abnormal and selective DNA methylation may constitute a potential biomarker and a tool to assess therapeutic treatments at the same time. The data on OS-mediated alterations in DNA methylation, which have been so far obtained, motivate chemoprevention trials, to reduce OS in cancer diseases [141–143]. In human aging, the telomerase reverse transcriptase (hTERT) controls the mitochondrial function and the cellular metabolism, besides the telomeres structure. The enzyme is regulated by DNA methylation. Various observations demonstrate that hTERT may confer major sensitivity towards OS [144] and reduce ROS increase in aging and age-related diseases [145]. Examples of both ROS levels and DNA methylation, which seems to change with age, suggest that they are potentially linked [146, 147]. ROS-induced methylation at SOD2 gene promoter causes the decreased expression of the gene, which may be associated with the disruption of the cardiorespiratory homeostasis, a typical problem of the old humans. Treatments with DNA methylation inhibitors, in preclinical studies, can prevent the hypoxic sensitivity that leads to the respiratory dysfunction [148]. Also, both ROS-induced 8-OHdG and 5-methyl cytosine generate abnormal GC regions in the DNA, which undergo further methylation and oxidation, thus hampering DNA repair enzymes. These regions have been demonstrated to hit gene expression and DNA susceptibility to damage in Alzheimer's pathology [149].

In complex, ROS are involved in DNA methylation processes in different conditions, occurring in the human aging. The epigenetic machinery operates as OS sensor, which contributes to the OS control and, at the same time, orchestrates the progressive homeostasis impairment, which shapes the cardiovascular, respiratory, and nervous systems of old human beings [146]. The ROS signaling in the DNA methylation during the aging process deserves to be more deeply studied.

## 5. ROS in Cell Senescence

The cell senescence has indicated the irreversible G1 growth arrest of normal primary cells, which occurs after the cells have accumulated time-dependent damage, during extensive culture passages (“replicative senescence”). The cells resist apoptosis and face malignant progression through cytostasis, thus causally contributing to cell senescence induction and maintenance. The senescent cells are able to diversify constantly, like cancer cells, but missing proliferation as a driver [7, 9]. Large and flat shape, rich cytoplasmic and vacuolar granularity, high levels of lysosomal *β*-galactosidase activity (SA-*β*gal), p16, p21, macroH2A, IL-6, phosphorylated p38MAPK, and “double-strand breaks” are the most common senescent cells features in* in situ* assays [9]. The exact mechanisms underlying the cell senescence onset and stabilization are still obscure. OS, mitochondrial deterioration, DNA damage, oncogenes expression, and loss of tumor suppressor genes, like PTEN, RB1, NF1, and INPP4, can induce cell senescence [9]. “Replicative senescence,” which is provoked by endogenous stimuli, is distinct from “stress-induced premature senescence,” which is provoked by exogenous stimuli. The two processes share molecular and functional features, although they are dependent, or not, on telomeres status, respectively. Intrinsic and extrinsic events can induce either the cell senescence or the apoptosis process, depending on the level of the impairment of the cell homeostasis [150] and the p53 activity [47]. The molecules secreted by senescent cells (secretoma) cooperate deeply to maintain the tissues homeostasis, through autocrine and paracrine activities [151], by acting at multiple levels: epigenome [152], gene expression, protein processing, and metabolic control [153]. Moreover, specific mitochondrial pathways contribute to priming the senescence process, through the alteration of the mitochondrial redox state [6, 151]. The senescence secretoma acts in physiological and pathological events, as tissue remodeling during embryogenesis, tissue repair in wound healing, and induction of aging, as well as age-related diseases of different organisms. The secretoma develops beneficial effects on carcinogenic DNA lesions of precancerous cells, by both preventing their uncontrolled cell proliferation and reacting with specific anticancer compounds [154]. However, the secretoma may provide indispensable cytokines for the cancer cells growth, thus promoting tumorigenesis in definite conditions, which are partly related to the cellular metabolic state [155]. Cause-effect relationships between cellular ROS production and cell senescence have been investigated through diverse pathways that comprise the following.

*(i) Mitochondrial DNA (mtDNA) Damage*. ROS contribute to cellular senescence onset and progression by damaging mtDNA directly or in synergy with modifications of the telomerase reverse transcriptase (TERT) enzyme and the p53 and Ras pathways activity [9]. Also, ROS production by serial signaling through GADD45-MAPK14 (p38MAPK)-GRB2-TGFBR2-TGFb is both necessary and sufficient for the stability of growth arrest, during the establishment of the senescent phenotype [156].

*(ii) Signaling Pathways via Ras, p53, p21, and p16*. The pathways generate ROS, which act as signaling molecules, without causing oxidative DNA damage. ROS result as a tightly regulated signaling process for the induction of the cell senescence [157].

*(iii) Autophagy*. High ROS levels mediate p53 activation that induces autophagy inhibition. This event generates mitochondrial dysfunction, which in turn generates cell senescence. The autophagy inhibition causes the senescent cells to aggregate oxidized proteins and protein carbonyls with products of lipid peroxidation and protein glycation into the lipofuscin [158].

*(iv) miR-210 and miR-494*. The induction of these microRNAs by ROS generates mitochondrial dysfunction and autophagy inhibition [159].

The (iii) and (iv) pathways generate vicious loop cycles in ROS production. Autophagy inhibition causes lipofuscin accumulation, which activates further autophagy impairment and ROS production, consequently. All the factors (i), (ii), (iii), and (iv) may add to DNA damage and dysfunctions of both mitochondria and cell metabolism homeostasis [159].* In vitro* and preclinical experiments show that ROS decreasing interventions influence cell senescence progression, via the slowdown of telomere shortening and the extension of the cell lifespan. Replicative telomere exhaustion, DNA damage, and OS prime the cell senescence by sharing the activation of the “DNA Damage Response.” ATM or ATR kinases of these signaling pathways cause p53 stabilization and transcriptional activation of the p53 target, p21 [9]. p53 triggers cell cycle arrest by upregulating p21, which inhibits the cell cycle regulator cyclin-dependent kinases Cdk4 and Cdk2 [159]. Whereas high OS levels induce the prosenescence function of p53, the mild OS levels that are induced by the physical exercise in humans have a positive effect on cell and mitochondrial homeostasis. p53 exerts a dual effect on cell senescence because of its ability to both decrease and increase the cellular OS level [160]. In parallel to “DNA Damage Response,” the mitochondrial p38-MAPK replenishes the short-lived DNA damage foci, via a ROS feedback loop, and induces the senescent secretoma [161].

The occurrence of the ROS role in cell senescence onset and maintenance might be relevant for therapeutic interventions, which aim to modulate ROS levels in cancer cells, as well as in aging processes [156]. Human kidney dysfunctions exemplify progressive stages of ROS-induced cell senescence. ROS act like a sensor in regulating the oxygen-dependent gene expression of the kidney and play a leading role in the inflammatory processes, to which the organ is especially sensitive [162]. In conclusion, the ROS signaling has highlighted key factors for the cell senescence induction and maintenance, which are the object of intensive investigations.

### 5.1. Cell Senescence in Aging and Age-Related Diseases (ROS Effect)

The “replicative cell senescence” is considered an aging hallmark on the basis of two motives: (1) the senescent cells accumulate in organismal tissues, by rate and proportion, which parallel the age advancement; (2) the senescent cells accelerate the age-related decrease of tissue regeneration, through the depletion of stem and progenitors cells [8, 97]. While the sequence of proliferative arrest (senescence), recruitment of immune phagocytic cells (clearance), and promotion of tissue renewal (regeneration) results in being beneficial upon a damaged tissue, for instance, the sequence is inefficiently completed in aging tissues, causing senescent cells to undergo chronic accumulation [163]. Also, a delicate balance exists between cell senescence positive effects on tumor suppression and negative effects on aging related processes [164]. The transcription factor and tumor suppressor p53 are involved in DNA repair and cellular stress response, as well as cellular cycle control. In addition, p53 modulates both the cell senescence and the aging process, through the coordination of specific cellular pathways [165, 166]. It is not clear whether p53 mechanisms in cell senescence and aging are common [160]. An increased senescence secretoma causes detrimental effects over the years and contributes to the typical disruption of aged tissues [8, 167, 168]. Senescent cells endowed with the semiselective marker of senescence p16 drive age-related pathologies, which are delayed or prevented by the selective elimination of the senescent cells [169]. A partial list of suggested markers of cell senescence in human tissues, both aged and affected by age-related pathologies, is reported in Table 2 [170–197]. Lungs show a typical example of cell senescence associated with the progressive, age-related organ dysfunction. The OS generated by the potent cigarette oxidants is a key element in the pathogenesis of the pulmonary emphysema, induced by the chronic smoking. The fibroblasts that provide essential support and matrix for lung integrity show reduced proliferation rate and increased SA-*β*gal activity in patients affected by pulmonary emphysema. These senescent fibroblasts contribute to the lung disease by affecting the tissue homeostasis. Also, senescent features of the endothelial cells in chronic smokers associate with premature vessels atherosclerosis. In patients with severe coronary artery disease, OS accelerates the senescence of endothelial cells, which is related to risk factors for cardiovascular disease [198]. A further example of aging dysfunction related to cell senescence is shown by the scaffolding protein Caveolin 1 (Cav1), which controls molecular signaling in caveolar membranes. Cav1 promotes cellular senescence in age-related pathologies, by mediating p53 activation with EGF modulation, focal adhesion, and small Rho GTPase-dependent signaling. The upregulation of the Cav1 promoter by high ROS levels contributes to explaining how OS promotes cell senescence effects in aging and age-related diseases [198]. In addition, the interplay between different conditions of mitochondrial homeostasis and ROS-dependent signaling pathways contributes to aging process, through the cell senescence induction and stabilization [199]. Yet ROS-independent signaling pathways link dysfunctions in mitochondria and aging, through the cell senescence process [6, 151]. As a new approach, preclinical and clinical studies demonstrate the therapeutic effects of the aging inhibitor rapamycin, whose signaling pathway is involved in cellular senescence [160, 200].

In conclusion, cell senescence reduces the age-related tumor development and contributes to human aging, suggesting that aging might be switched for tumorigenesis [201, 202]. ROS may modulate tumor suppression process, which is induced by the senescence, thus participating in anticancer mechanisms, although ROS may act as tumor promoters in definite conditions [48]. With the cell senescence and aging controlled by cells and cellular environment, the possibility is suggested that the two processes may be subjected to interventional therapies [203, 204].

### 5.2. Epigenetic Mechanism in Cell Senescence (ROS Involvement)

The epigenetic control of acute and chronic cellular senescence allows for the two processes that are involved in various conditions that lead to the cells longevity preventing cell death and tumorigenesis [205]. The abrogation of tumor suppressor pathways, as p53 and p16/Rb, bypasses the cell senescence, thus leading to the tumorigenic phenotypes acquiring [206]. The mechanisms that balance the transcriptional state of the chromatin are not fully understood. Some regulative changes involve the histone proteins that coordinate the DNA accessibility, through transcription factors, besides the DNA replication and repair. The Polycomb Repressor Complex 2 (PRC2) initiates and preserves specific histone methylations, thus acting as an epigenetic mark that mediates targeted genes [207]. The repression of the histone activity by the Polycomb Group (PcG) proteins causes gene silencing, but it can be countered by specific demethylases, which erases the methyl mark [208]. The upregulation of many PRC target genes leads to global epigenetic changes [209–211]. Specific transcription factors [212], as well as long noncoding RNAs [213], are involved in the recruitment performed by PRC. PRC2 takes a crucial part in silencing the locus of p16, the marker that is upregulated during cell senescence [212]. The reversal of chromatin epigenetic pattern via deacetylation, demethylation, and dephosphorylation is significantly involved in underscoring both flexible and dynamic nature of histone modifications [214]. The histone demethylases JMJD3 produce diverse outputs of biological function, depending on the action of their transcriptional complexes. Different expression of these demethylases, which have tumor suppressor activities during the “stress-induced senescence” [215, 216], is reflected into cellular phenotype changes and variations associated with cellular senescence [217]. The JMJD3 gene is located near the p53 tumor suppressor gene, that is, a genomic area that is frequently lost in various malignancies. The SIRT1 histone deacetylase (SIRT1) is a known regulator of age-related diseases that regulates the senescence secretoma components, by silencing their promoter regions epigenetically. SIRT1 plays a pivotal role in stress modulation also through p53 deacetylation, acting against aging and age-related diseases. As indicated above, the high ROS levels activate p53, which, in turn, activates p53-mediated apoptosis and cell senescence. Moreover, SIRT1 regulates the ROS-dependent FOXO factors, which are responsible for cell growth, proliferation, and longevity. The characteristic ROS increase during aging may be responsible for the decreased SIRT1 activity, which facilitates the senescent-like phenotype. SIRT1 causes oxidant effects, as well as antioxidant effects, by acting on epigenetic modifications, which include acetylation and deacetylation (see references in [128, 146]). Experiments on cell senescence induction show different molecular mechanisms in acute versus chronic senescent cells. A better knowledge of the order in which epigenetics mechanisms change during the cell senescence progression, from initial towards full senescence, is believed to be vital for finding therapies against age-related disorders [9].

#### 5.2.1. Noncoding RNA

Latest genomics tools and sequencing approaches have helped unravel large chromosomes stretches, which were previously deemed not transcribed [218, 219]. These sequence regions contain noncoding RNA (ncRNA), which is known as long lncRNAs, and short ncRNAs. Among short ncRNAs, the microRNAs (miRNAs) have emerged as being able to control the gene expression, either by blocking targeted mRNA translation or by mRNA degrading [220, 221]. Recently, ncRNA role is gaining more importance in age-associated dysfunctions as cardiovascular diseases [222, 223]. The senescence-associated lncRNAs are differentially expressed in proliferating and senescent fibroblasts, as assessed by RNA sequencing [224–226]. Toxicological studies associate increased ROS production with increased expression of a set of 115 lncRNAs, which significantly affect p53 signaling pathway [227]. A mitochondrial-transcribed lncRNA is induced in aorta and endothelial cells aging, during the “replicative vascular senescence,” which is partly responsible for age-associated cardiovascular diseases, but not in the “stress-induced premature senescence” by ROS [228].

#### 5.2.2. microRNA (miRNA, miR)

Normal cellular development and homeostasis are under the control of miRNAs, throughout the entire life [229], since miRNAs regulate the gene expression in biological processes as proliferation, development, differentiation, and apoptosis. Yet several miRNAs families control cell senescence at multiple levels, by regulating the autophagy process and the gene expression involved in ATP and ROS production. Some miRNAs may induce ROS production that generates a self-sustaining ROS vicious cycle [159]. miRNAs constitute a connection between aging, cell senescence, and cancer. The miRNAs dysregulation causes the activation of pathways they normally repress. The event may activate aberrant pathways and also aging mechanism in young individuals [222]. Although current studies are monitoring miRNA tissues and systemic alterations, instead of miRNA changes through lifespan and metabolic modifications, several profiles of miRNA expression demonstrate changes during the aging. As an example, miR-29, which targets the genes of type IV collagen and maintains the structure of the extracellular matrix, increases in elderly mice, thus causing collagen decreasing, a tissues basement membranes weakening [230]. Only few miRNAs have been directly linked to age-related changes in cellular and organ functions, whereas many miRNAs have been directly connected with disease states. It is unclear if the modifications of miRNA profiles are mostly involved in pathological changes onset or if they mark the senescence end, which leads to the organ aging and dysfunction. Altered expression in miRNA activity has been observed in elderly people, as in the case of miR-34a, which belongs to a family with conserved functions in controlling aging and age-related diseases [203, 231, 232]. miR-34a targets ROS scavenger enzymes inducing OS [159]. The miR-34a upregulation or overexpression has been associated with cell proliferation inhibition, subsequent cell senescence induction, and premature death, in both endothelial progenitor and mature cells. miR-34a causes memory function impairment when it is upregulated in aged mice and in models for Alzheimer's disease (AD), while miR-34a targeting restores the memory function [233]. Also, the miR-34 mutation of the loss-of-function delays the age-related decline markedly, thus resulting in extended lifespan and increased resistance to the heat and the OS. The human miR-34a is downregulated in Parkinson's disease brain, while it is upregulated in AD brains [234] and in plasma of Huntington's disease patients [235].

**Figure 2:**
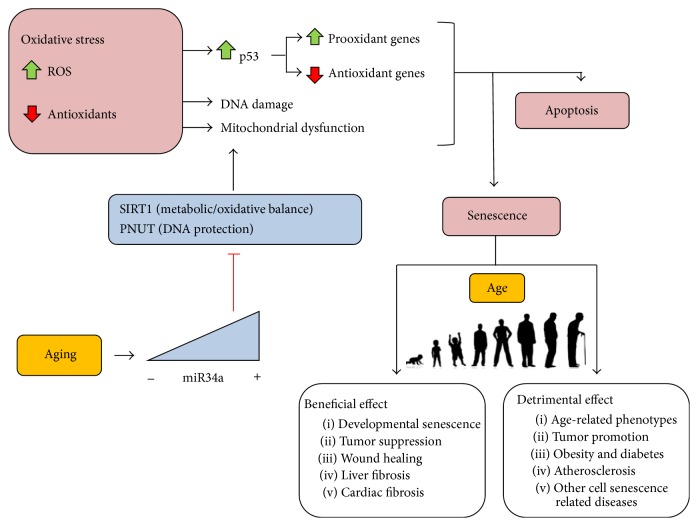
ROS-mediated senescence. Besides causing DNA damage and mitochondria dysfunction, OS activates p53 that, in turn, induces prooxidant genes and imbalances antioxidant genes induction. The set of alterations caused by ROS lead to induction of cell senescence, which, in turn, can develop both positive and negative effects; miR34a expression increases with aging in many tissues downregulating SIRT1 protein activity (a longevity promoting factor) and PNUT protein (a DNA protecting factor which prevents telomere attrition and is involved in tissues repairs).

Several miRNA families are modulated by ROS in the development of mitochondria-mediated cell senescence, which are, indirectly or directly, implicated in human pathologies. Little is known about the roles of ROS-modulated miRNAs in cell function. The molecular mechanisms that control neuronal response to OS have been deeply studied in different strains of senescence accelerated mice, based on the consideration that OS plays a critical role in AD etiology and pathogenesis. OS upregulates a group of miRNAs (miR-329, miR-193b, miR-20a, miR-296, and miR-130b), which is associated with affecting 83 target genes. Among the genes, mitogen-activated protein kinase signaling pathway has been suggested to play a role in pathogenesis of neurodegenerative diseases [233]. OS effects on vascular homeostasis, including angiogenesis in physiological processes and age-related diseases, are largely studied in human umbilical vein endothelial cells (HUVECs), considering that miRNAs modulate endothelial cells response to OS. ROS induce the expression of miR-200 family members (miR-200c, miR-141, miR-200a, miR-200b, and miR-429), which determines apoptosis and cell senescence both in HUVEC cells and in a model of hind limb ischemia, which shows OS-mediated mechanism [236]. The miR-200 family plays a causative role in the vascular diabetic inflammatory phenotype in a diabetic model and in the human vasculopathy disease, suggesting that miR-200 inhibition might represent a therapeutic target to prevent OS negative effects on cell function and survival [146]. Also, miR-200 family has been extensively studied in epithelial-to-mesenchymal transition of cancer cells [236]. Lately, miR-760 and miR-186 upregulation has been associated with replicative senescence in human lung fibroblast cells. These miRNAs cooperate to induce senescence through the ROS-p53-p21Cip1/WAF1 pathway, which depends on the ROS generated by the downregulation of the protein kinase 2 (CK2*α*). A better understanding of the mechanisms of CK2 regulation might provide new therapeutic options to restore the function of lungs in aged people. An example of the increasing evidence that miRNAs are critically involved in the posttranscriptional regulation of cell functions, including the ROS signaling modulation, is underlined in Figure 2.

## 6. Conclusion and Future Perspectives

The multifactorial and inexorable phenomenon of aging worsens the human functions at multiple levels, causing a gradual reduced ability to resist stress, damage, and illness. Healthy aging appears to be an ideal healthcare priority that entails a better understanding of aging, with the aim of slowing down the process and preventing or even treating its related pathologies [200]. Indeed, genetic insights combined with findings from animal and cellular models have advanced our understanding of pathways that lead to age-related features, highlighting possible interventional targets [2–5]. The cellular senescence process is considered an aging hallmark, because it drives the cells through longevity, by hampering tumorigenesis and cell death, and is involved in many age-related diseases [97, 205, 206]. The cell senescence is a feature that characterizes somatic cells, except for most tumor cells and certain stem cells [6–10]. The senescent cells produce a specific secretoma that cause beneficial effects, through its autocrine and paracrine mechanisms. When the senescent cell program is inefficiently developed, as it occurs during the aging, the secretoma causes detrimental effects [151–153, 167, 168, 199]. In the recent years, evidence has been accumulating that ROS, which include H_2_O_2_, superoxide, anion, and hydroxyl radicals, generated from both intrinsic and extrinsic events, inhibit cell growth and induce cell death and senescence in a context-dependent manner [157, 236]. Through the understanding of the ROS role as signaling molecules in a myriad of signaling pathways, ROS levels are no longer considered as mere metabolic byproducts but are believed to be a “redox biology” that regulates physiological functions, including signal transduction, gene expression, and proliferation [37]. Firstly, it has been evidenced that the DNA damage caused by ROS acting as mutating agents contributes to the induction and maintenance of the cell senescence process [9, 156]. More recently, particular attention has been focused on the ROS involvement as signaling molecules in cell senescence induction, without causing DNA damage. Signaling pathways via Ras, p53, p21, and p16 have been defined to generate ROS, which may act as tightly regulated process contributing to the cell senescence induction [20, 157, 158]. Cause-effect relationships between cell ROS production and cell senescence have been investigated through diverse pathways that include the field of mitochondrial DNA and autophagy inhibition and the effects of the microRNAs miR-210 and miR-494 in various mitochondrial processes [159]. These pathways highlight ROS contribution to prime cell senescence at diverse levels, among which epigenetic level is attracting more and more attention in studies aimed at the senescence control [227, 233, 236]. Indeed, the epigenetic modulation provides the essential and flexible interface between the organisms and the environment, which results in being essential for all the cell functions [122, 123, 129], throughout the lifespan [135–137]. A major breakthrough in the last decades has been the understanding that epigenetics contribute to human diseases development.

In parallel, the “OS theory of aging” remains the most documented mechanistic hypothesis of aging, although it does not necessarily imply ROS imbalance as the earliest trigger or the main cause of aging [98–103]. Therapeutic ROS modulation is suggested as relevant in aging and related events [95, 96, 114]. Also, the senescent cells have been identified as a novel potential therapeutic target in the aging and age-related diseases [169, 171]. Further research is needed to define when and where cell senescence results in being favorable or unfavorable to organismal health. Both pro- and antisenescent therapies can be equally helpful, when they are opportunely modulated and balanced. Prosenescent therapies contribute to minimize damage in the cancer disease and in the active tissue repair by limiting proliferation and fibrosis, respectively, while antisenescent therapies may help to eliminate accumulated senescent cells and to recover tissue function. The current research points to a double objective: to define the changes about the redox-sensitive cell pathways and to define the OS role in linking environmental factors with epigenetic modifications.

Particular emphasis is addressed to novel mechanism of ROS and epigenetics in cell senescence and aging [160, 165, 166]. The histone demethylases network is often synergizing with the action of histone deacetylases, histone methyl transferases, and various nuclear transcriptional complexes, thus ensuring that the chromatinic environment is correct for the cell [128, 146]. Preclinical and clinical examples of ROS-dependent epigenetic modifications [125–127, 130–134, 138] extend their effects to aging [135, 136] and age-related diseases [137, 142–144, 146–149], particularly towards cancer disease [139–141, 145]. Among the noncoding RNAs, miRNAs families provide a broad silencing activity of mRNA targets, in a sequence dependent fashion that modulates the stress response [159]. Accumulating evidences show that stressors, including ROS, potentially alter the function of miRNA-processing in aging organisms, which renders the cells even more prone to stress, linking aging and cancer. Several miRNAs families induce ROS level increase in aging or target factors involved in the ROS signaling. In addition, ROS increase highly correlates with a specific miRNA dysregulation, which mediates the cross talk between p53, NF-*κ*B p65, and ROS. All these events have been associated with cell senescence [203, 231, 232]. At the same time, certainly several miRNAs families are modulated by ROS in the development of mitochondria-mediated cell senescence, which are, indirectly or directly, implicated in human pathologies [159, 233, 236]. Because epigenome is so tightly regulated and complex, understanding individual modifications and their network of interaction offers the potential to design drugs that are very effective therapies against a number of diseases [124, 203–205, 219–222]. More reliable OS biomarkers, as well as OS related epigenetic mechanisms, have emerged over the last years as potentially useful tools to design therapeutic approaches aimed at modulating* in vivo* enhanced OS.

## Figures and Tables

**Table 1:** Selected ROS sensitive proteins that are involved in cell signaling transduction mechanism. Indicative examples of possible effects and processes they promote after being directly and/or indirectly modified by ROS (the references are indicated inside the square brackets).

ROS sensitive proteins: *oxidative interface*	(1) Effects of ROS sensitive proteins after being redox modified	(2) Physiopathological processes in which ROS sensitive proteins are involved
*Protein kinases*		
Receptor/nonreceptor tyrosine kinases (Src, TRK, AKT, c-Abl, MAPK, CaMKII, PKG, ATM, and Ask1)	(i) Interactions between kinases pathways [38, 39] (ii) Signal of ROS production feedback [40]	Control of cell cycle progression [56] Mitosis for anchorage-dependent cells [57] Cellular homeostasis [43, 57]
AMP-activated protein kinases (AMPK)	(i) Regulation of cell ROS/redox balance [41, 42]	Myocyte adaptation to energy requirement [42] Adipocyte differentiation [58] Lipid metabolism (“fatty liver”) [59] Hyperglycemic damage [60] Cell fate (autophagy and apoptosis) [61]

*Adaptor proteins*		
p66Shc	(i) Signaling start in the aging process [43]	Apoptosis [43]. Prolonged life span [43, 62] Cardiovascular diseases and obesity [63] Diabetic endothelial dysfunction [64]

*Nuclear receptors*		
PPAR*γ*	(i) Redox sensor function [43] (ii) Regulation of genes that modulate ROS increases [44]	Neurodegenerative diseases [65, 66] Lipid dysfunction (fatty liver) [59]

*Membrane receptors*		
Elements in Notch1 pathway	(i) Notch signaling modulation in association with Wnt/beta-catenin signal [45]	Cell fate control in vascular development [45] Biological clocks in embryonic development [67]

*Transcription factor*		
p53	Modulation of cell redox balance (prooxidant/antioxidant effects) [46–48]	Cell fate signaling [68] Autophagy and apoptosis [61, 69]
Nrf2	Cell adaptation to ROS resistance [49, 50]	Apoptosis [70] Neurodegenerative diseases [71] Cardiovascular diseases [72]
FOXO3A	Cell coordination in response to OS [51]	Metabolic adaptation to low nutrient intake [73] Cancer development [73] Diabetes [74] Atherosclerotic cardiovascular disease [75]
Components in *β*-catenin/Wnt pathway	Regulation of Wnt signaling via nucleoredoxin [76]	Early embryonic development [76] Vascular development [45]
HIF-1a	Cell adaption to oxygen tension modifications [52]	Cell proliferation; angiogenesis [77] Cell transformation [78, 79]
Components in JAK–STAT pathway	(i) Cell adaption to OS [53] (ii) Mediation of ROS mitogenic effect [53]	Stress response gene expression [51] Systemic/pulmonary hypertension [80]
NF-*κ*B	Regulation of redox-sensitive gene expression [54, 55]	Rheumatoid arthritis, dyslipidemia, atherosclerosis, and insulin resistance [81]

**Table 2:** Clinical examples of senescence-associated biomarkers detected in organs and tissues of patients affected by age-related diseases.

Organ/tissue	Senescence-associated biomarkers	Clinical references
*Cardiovascular diseases*		
Aged vascular tissues	Telomeres length, SA-*β*Gal, p16, and p21	[170, 171]
Atherosclerosis		
Systolic heart failure		
*Malignant tumors*		
Lung cancer	Telomeres length, SA-*β*gal	[172, 173]
Breast cancer	SA-*β*gal, p21, p16, DEP1, NTAL, EBP50, STX4, VAMP3, ARMX3, B2MG, LANCL1, VPS26A, and PLD3	[174, 175]
Neuroblastoma	SA-*β*gal	[176]
Astrocytoma	SA-*β*gal	[177]
Mesothelioma	SA-*β*gal, p21	[178]
Melanoma	SA-*β*gal, p16, and p21	[179]
Prostate cancer	SA-*β*gal, Glb1, and HP1g	[154, 180]
Liver cancer	Telomeres length, SA-*β*gal	[181]
Colorectal cancer	Short telomeres	[182]
*Fibrosis*		
Idiopathic pulmonary fibrosis	Telomeres length, IGFBP5, and SA-*β*gal	[183, 184]
Cystic fibrosis	Telomere length, p16	[185]
Liver fibrosis	Telomere length, IGFBP-5, SA-*β*-gal, and p21	[183, 186]
Renal fibrosis	p16	[187, 188]
*Neurological disorders*		
Alzheimer's disease	SA-*β*-gal	[189, 190]
*Other diseases*		
Chronic obstructive pulmonary disease	Telomere length, p16, p21, and SA-*β*gal	[191, 192]
Pulmonary hypertension	p16, p21	[192, 193]
Emphysema	Telomere length, IGFBP-3, IGFBP-rP1, p16INK4a, and p21	[194, 195]
Benign prostatic hyperplasia	SA-*β*gal	[196, 197]

## Abbreviations

AP-1: Activator protein-1
DDR: DNA Damage Response
FOXO3a: Forkead homeobox type O
HIF-1a: Hypoxia inducible factor-1a
hTERT: Human telomerase reverse transcriptase
miRNA, miR: MicroRNA
JAK/STAT: Janus kinase/signal transducers and activators of transcription
Nox: NADPH oxidases
NF-*κ*B: Nuclear factor kappa B
NS: Nitrosative stress
Nrf2-ARE: NF-E2-related factor 2 binding to the antioxidant responsive elements
p53: Tumor suppressor p53
OS: Oxidative stress
PPAR*γ*: Peroxisome proliferator-activated receptor gamma
RNS: Reactive Nitrosative Species
ROS: Reactive Oxygen Species
SA-*β*gal: Senescence-associated *β*-galactosidase
SOD: Superoxide dismutase.
